# Supplementary figures and images for: Involvement of glycogen metabolism in circadian control of UV resistance in cyanobacteria
Source: PLoS Genet. 2020 Nov 30;16(11):e1009230. doi: 10.1371/journal.pgen.1009230 (PMC7728383; doi:10.1371/journal.pgen.1009230)

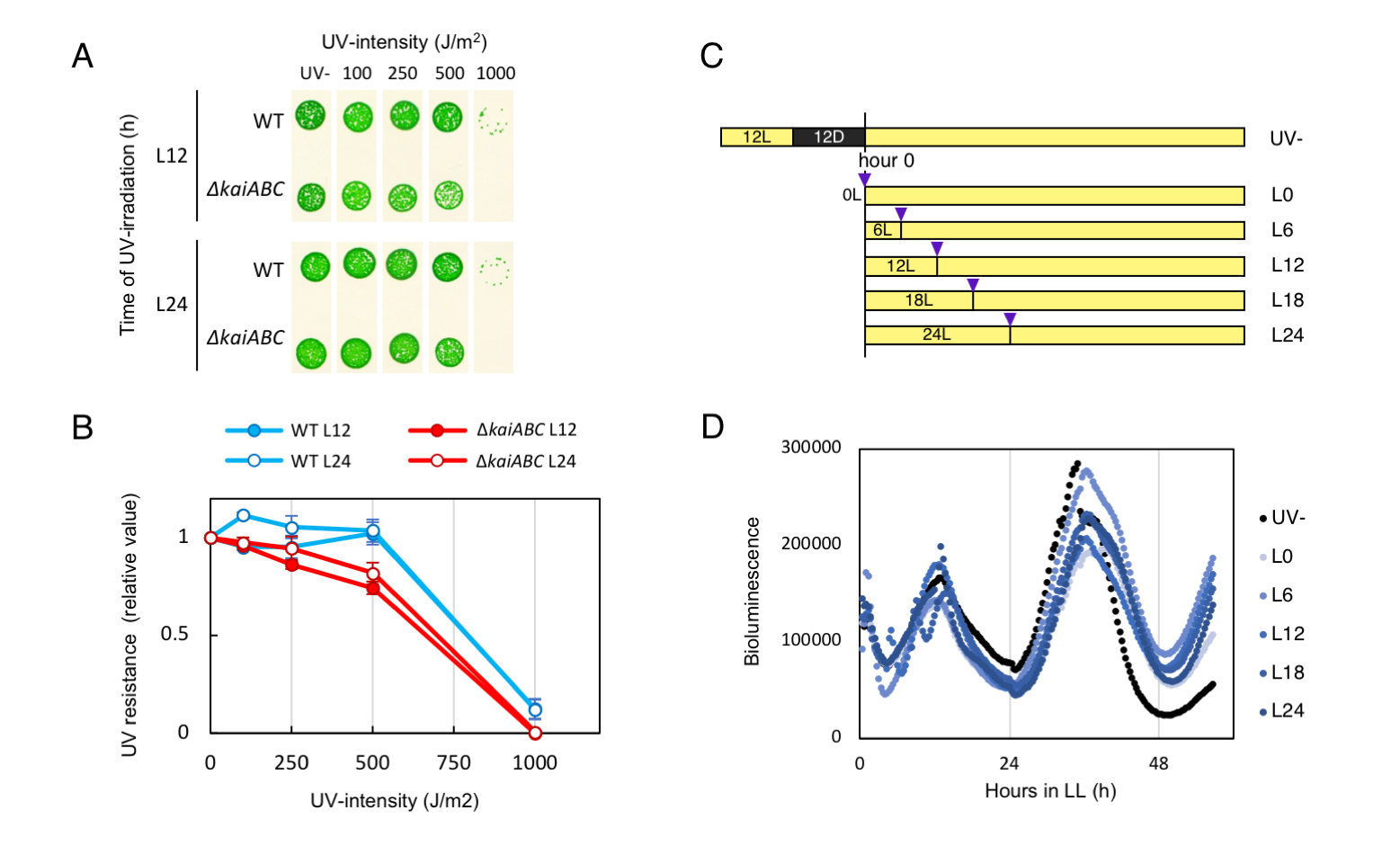

Supplement: S1 Fig — (A) UV-dose dependency under the UV+L condition. Growth of the UV-C-irradiated cells. Each image represents a spot assay of cellular growth after UV irradiation of 0–1000 J/m2 at hour 12 or 24 in the light (L12 or L24) under the UV+L condition (as shown in Fig 1D). Representative data of three independent experiments are shown. (B) Densitometric analysis of the growth test in S1A Fig. The intensity of UV irradiation is shown on the horizontal axis, and relative UV resistance is shown on the vertical axis, as in Fig 1C (n = 3). Error bars indicate the standard deviation. (C) Phase responses against UV-C Irradiation. A schematic representation of the experimental schedule. After the cells were synchronized to two 12-h:12-h LD cycles, bioluminescence rhythms were monitored to measure the kaiBC promoter activity under LL condition. Cells were subjected to UV-C irradiation at each time point (arrowheads). (D) Bioluminescence rhythms of cells subjected to UV-C irradiation at the indicated time points. The results indicate the UV-C irradiation did not affect the phase of the endogenous oscillator under our experimental conditions. (TIF) [file pgen.1009230.s001.tif]

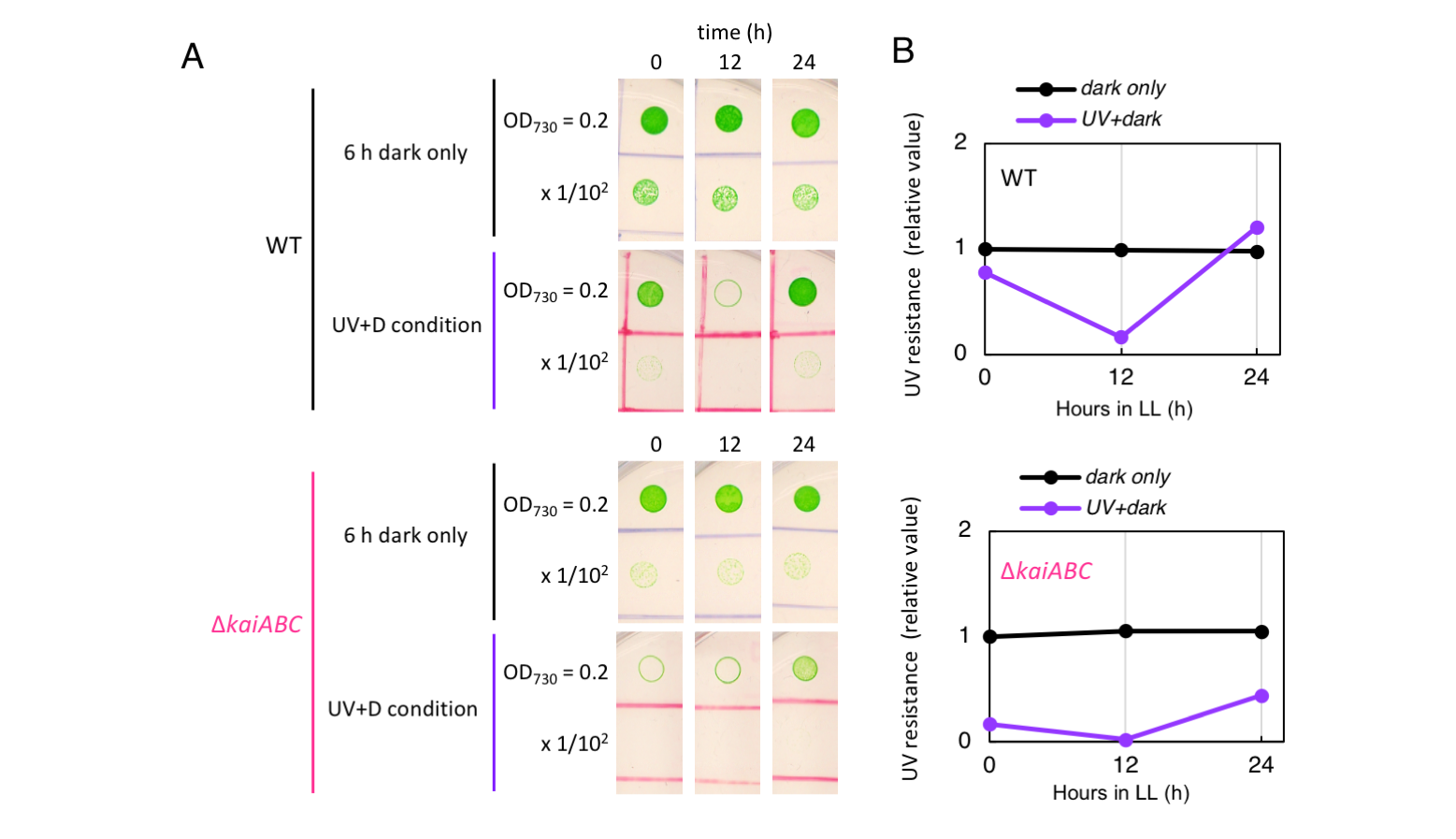

Supplement: S2 Fig — (A) Growth of the cells under UV+D condition or “6-h darkness only” condition without UV-C irradiation (WT, wild-type; ΔkaiABC, kaiABC-deficient strain). Spot tests were performed with cell suspension with an optical density at 730 nm (OD730) of 0.2 and 0.002. (B) Densitometric analysis of the growth test in S2A Fig (using the results for spots starting from cell suspension with OD730 of 0.2). The value for dark-exposure-only sample at hour 0 for each strain was normalized to the value of 1. (TIF) [file pgen.1009230.s002.tif]

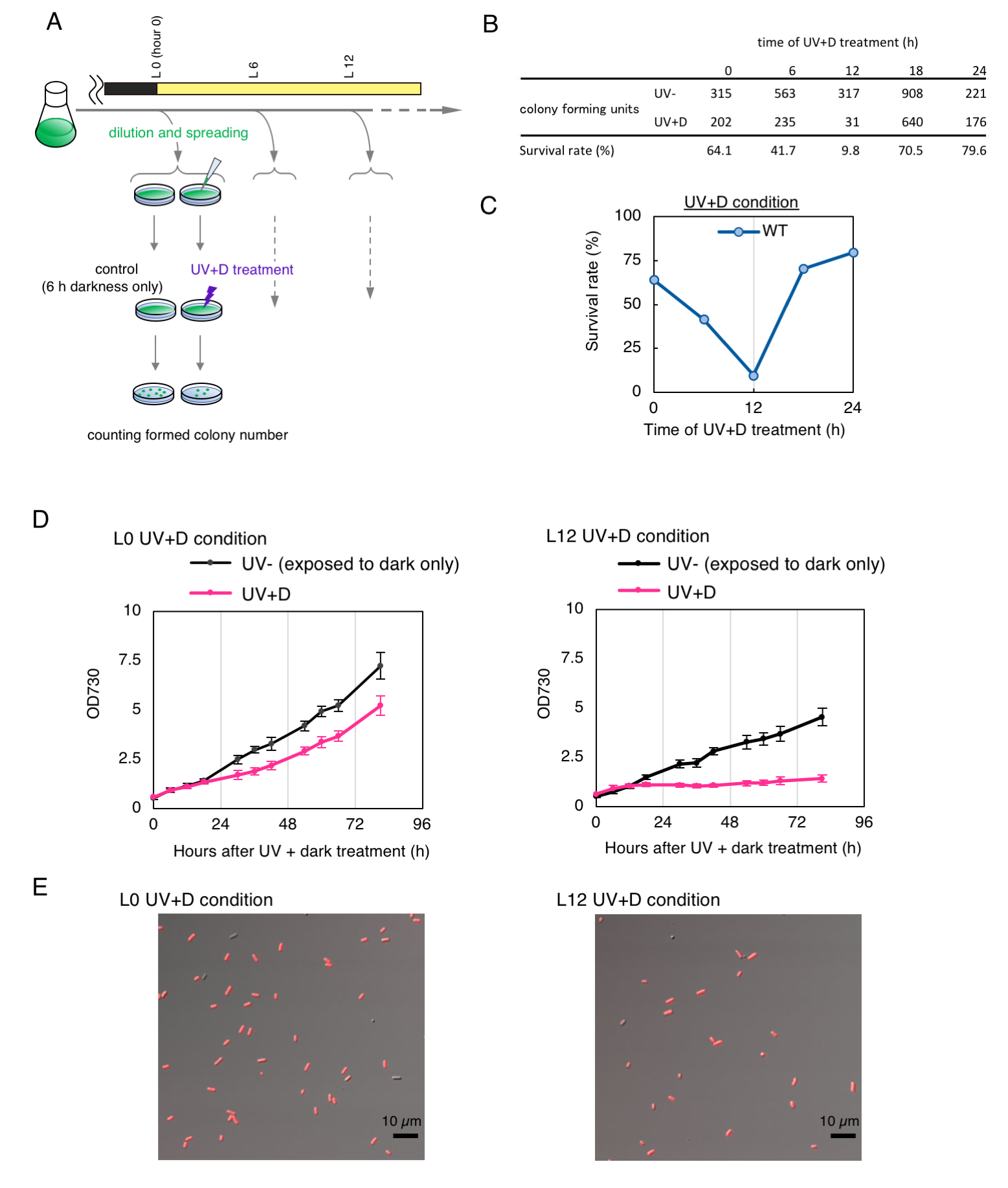

Supplement: S3 Fig — (A) Circadian variation of survival rates after UV-C irradiation under the UV+D condition. A schematic representation of experiment. WT cells were cultured and synchronized to a 12-h:12-h light:dark (LD) cycles in BG-11 liquid media, then plated on agar media at each time point, and subjected to UV irradiation under the UV+D condition. The plates were further incubated under continuous light. (B and C) The survival rates of UV-C irradiated WT cells under the UV+D condition. Survival rates were calculated by counting the colony-forming units and normalized to UV(–) control samples. (D) Time dependent reaction to UV Irradiation in liquid culture. Growth of the UV-C irradiated WT cells under the UV+D condition. UV irradiation was performed at hour 0 or 12 in LL. The growth curve in each condition was shown (left panel, L0; right panel, L12). Time after UV irradiation and subsequent darkness are shown on the horizontal axis. OD730 of cell culture is shown on the vertical axis (UV–; negative control without UV irradiation). (E) Images of UV irradiated cells. Cells were sampled from the experiments shown in S3D Fig at 60 h after UV+D treatment. Images show an overlay of differential Interference contrast (gray) and autofluorescent (red) microscopic pictures. Autofluorescence signals of cells due to photosystems were obtained (for details, see Materials and Methods). (TIF) [file pgen.1009230.s003.tif]

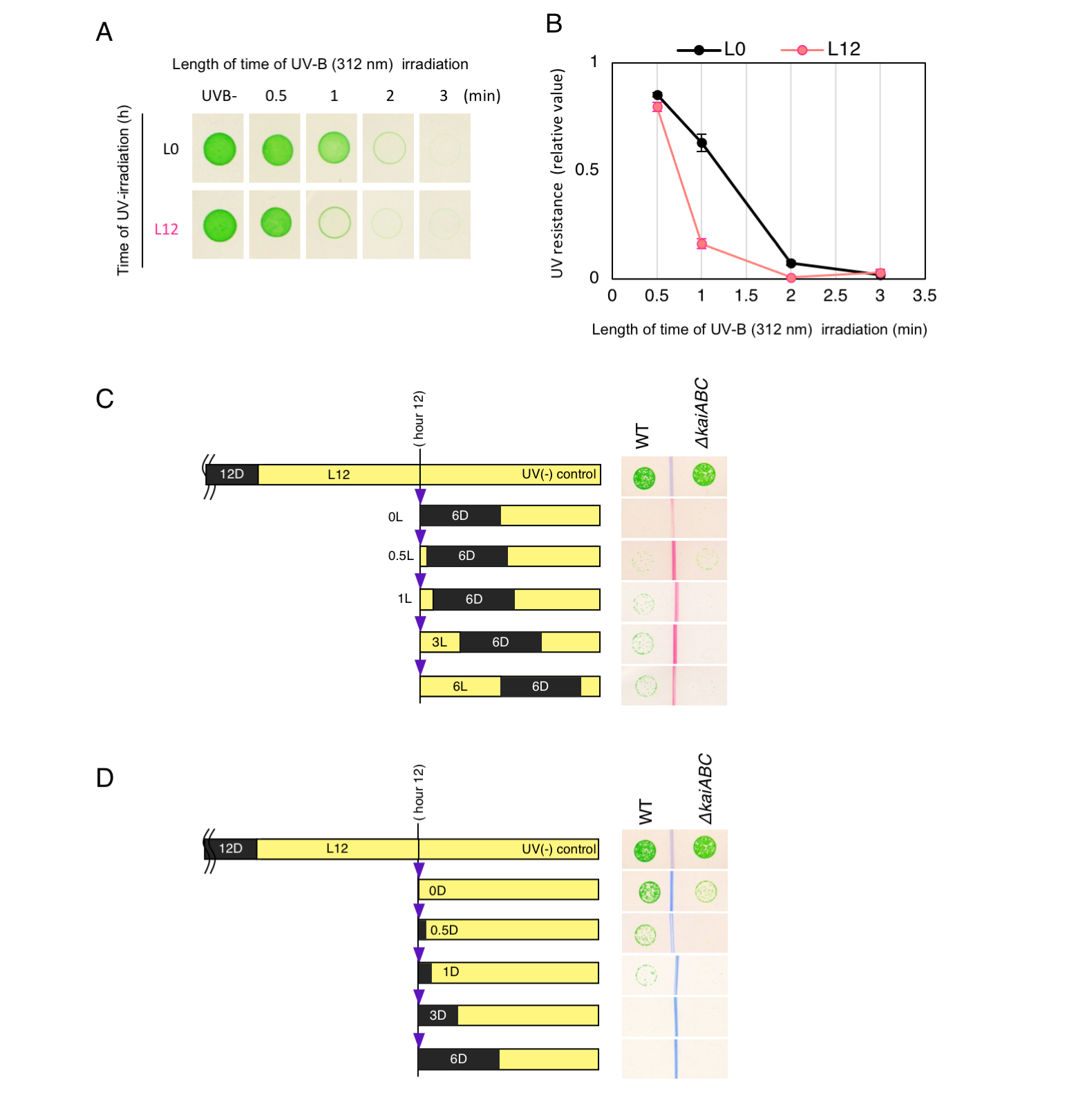

Supplement: S4 Fig — (A) Time-dependent response to UV-B irradiation. Growth of the UV-B irradiated cells. UV irradiation was performed using a discharge tube with a main wavelength of 312 nm (UV-B intensity was 5.22~5.88 mW/cm2). Each photo represents spot-plate growth following UV-B irradiation. Left label represents the time of UV irradiation and upper label represents durations of UV-B exposure. (B) Densitometric analysis of the growth test in S4A Fig. UV resistance was normalized to the densitometric value of a corresponding negative control strain without UV exposure. Error bars indicate the standard deviation. (C) The effects of changing light periods in response to UV-C irradiation at hour 12 in the light. A schematic representation of the experimental schedule is shown on the left, and the growth of the UV-C-irradiated cells is shown on the right. Each schedule on the left side is arranged to correspond to the experimental results. Representative data of three independent experiments are shown. (D) Effects of changing dark periods after UV irradiation at hour 12 in the light under the UV+D condition. Each symbol or image is the same as in S4C Fig. (TIF) [file pgen.1009230.s004.tif]

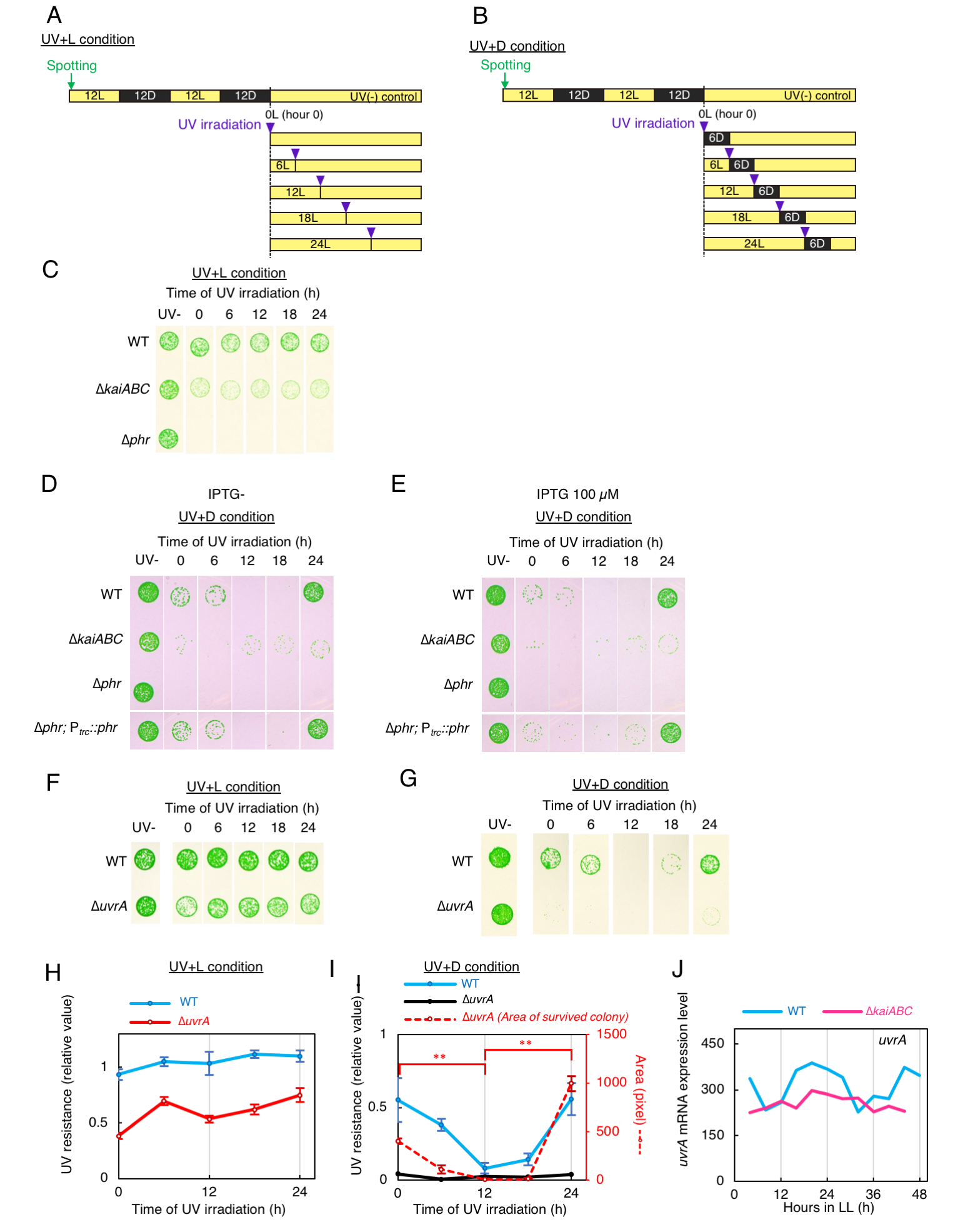

Supplement: S5 Fig — (A and B) A schematic representation of the experimental schedule. Each symbol is the same as in Figs 1D and 2A. (C) UV-C resistance of the Δphr strain under the UV+L condition. Spot assay to assess growth of the UV-C-irradiated cells. UV irradiation was administrated at each indicated time under the UV+L condition. (D and E) Effect of the addition of IPTG to Δphr; Ptrc::phr strain under the UV+D condition. S5D Fig and Fig 3A are based on the same experimental design, while the images were obtained from independent experiment as the IPTG- negative control against the IPTG+ data shown in S5E Fig. Each image represents a spot assay to assess growth under the UV+D condition as shown in Fig 2B. Experiments were performed in the absence of IPTG (D) or the presence of 100 μM IPTG (E). (F and G) UV-C resistance of the ΔuvrA mutant under each experimental condition. Growth of UV-C-irradiated cells (WT; ΔuvrA, uvrA-deficient strain). Each image represents a spot assay to assess growth following UV irradiation at each time point under the UV+L condition (F) and the UV+D condition (G). (H and I) Densitometric analysis of the growth test in S5F Fig (H) and S5G Fig (I). Since colony formation was severely suppressed in the uvrA strain, it was difficult to obtain appropriate values when applying the usual densitometry analysis due to the effect of background noise. Therefore, the area of the colony was shown as a dashed red line to validate its rhythmicity. Values of the colony area differed significantly between hours 0 and 12 and between hours 12 and 24 (** P< 0.01, Student’s t-test), supporting the rhythmicity. (J) uvrA mRNA accumulation profile in each mutant strain. The uvrA expression level from microarray data reported in the previous studies is shown. The microarray data were extracted for ΔkaiABC and the corresponding control wild type (WT) strains from Ito et al. [14]. (TIF) [file pgen.1009230.s005.tif]

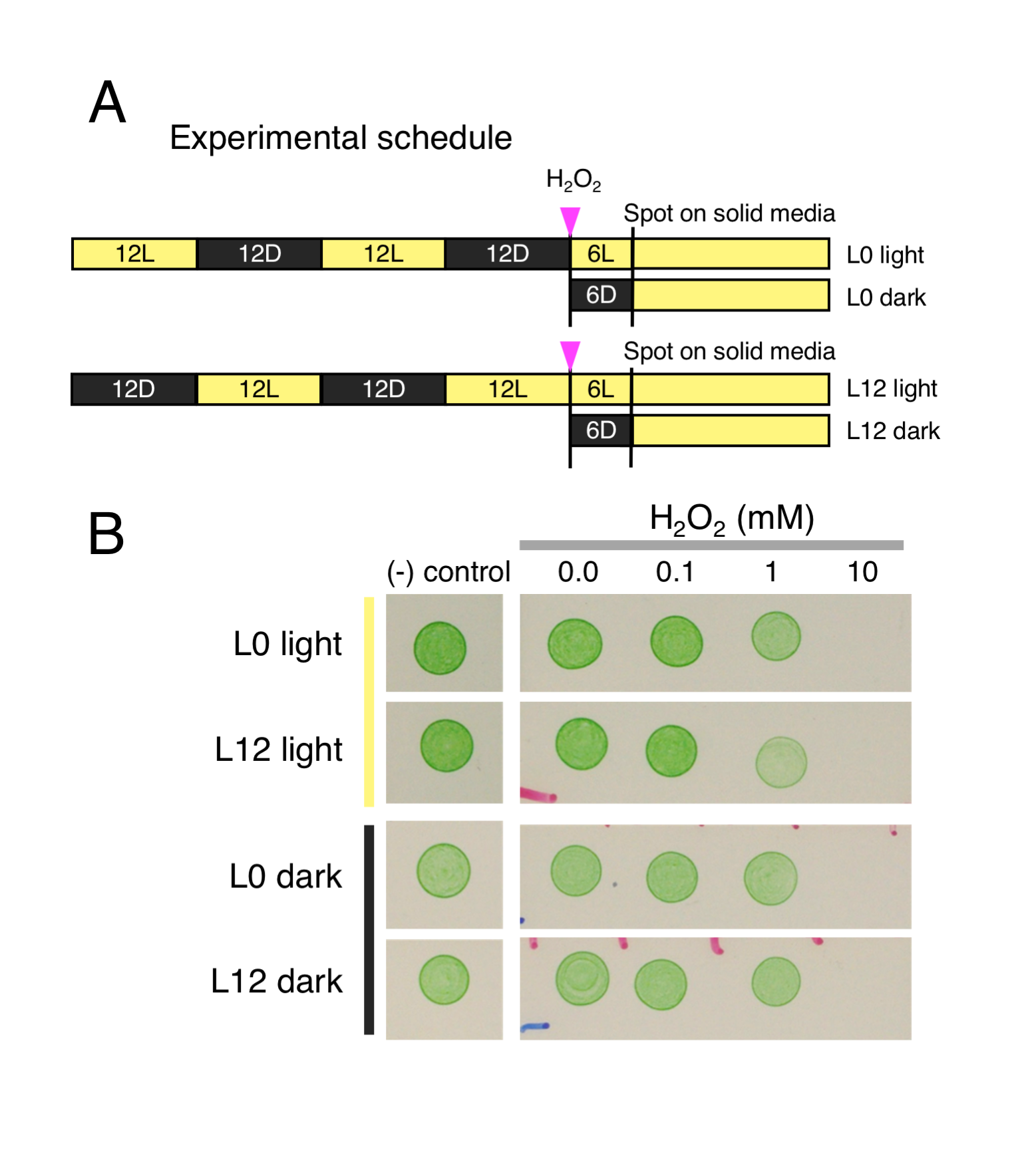

Supplement: S6 Fig — (A) A schematic representation of the experimental schedule. After the cells were synchronized to two 12-h:12-h LD cycles, H2O2 was added to the liquid cultures at hour 0 or 12 in LL, respectively (arrowhead). Following the addition of H2O2, each cell suspension was exposed to light or dark for 6 h, then spotted onto solid media and cultured under the LL condition. (B) Each image represents a spot assay to assess growth following the addition of H2O2. Upper panels represent the final concentration of H2O2 ((–) control; negative control without H2O2). Light and dark on the left side represent conditions after the addition of H2O2. (TIF) [file pgen.1009230.s006.tif]

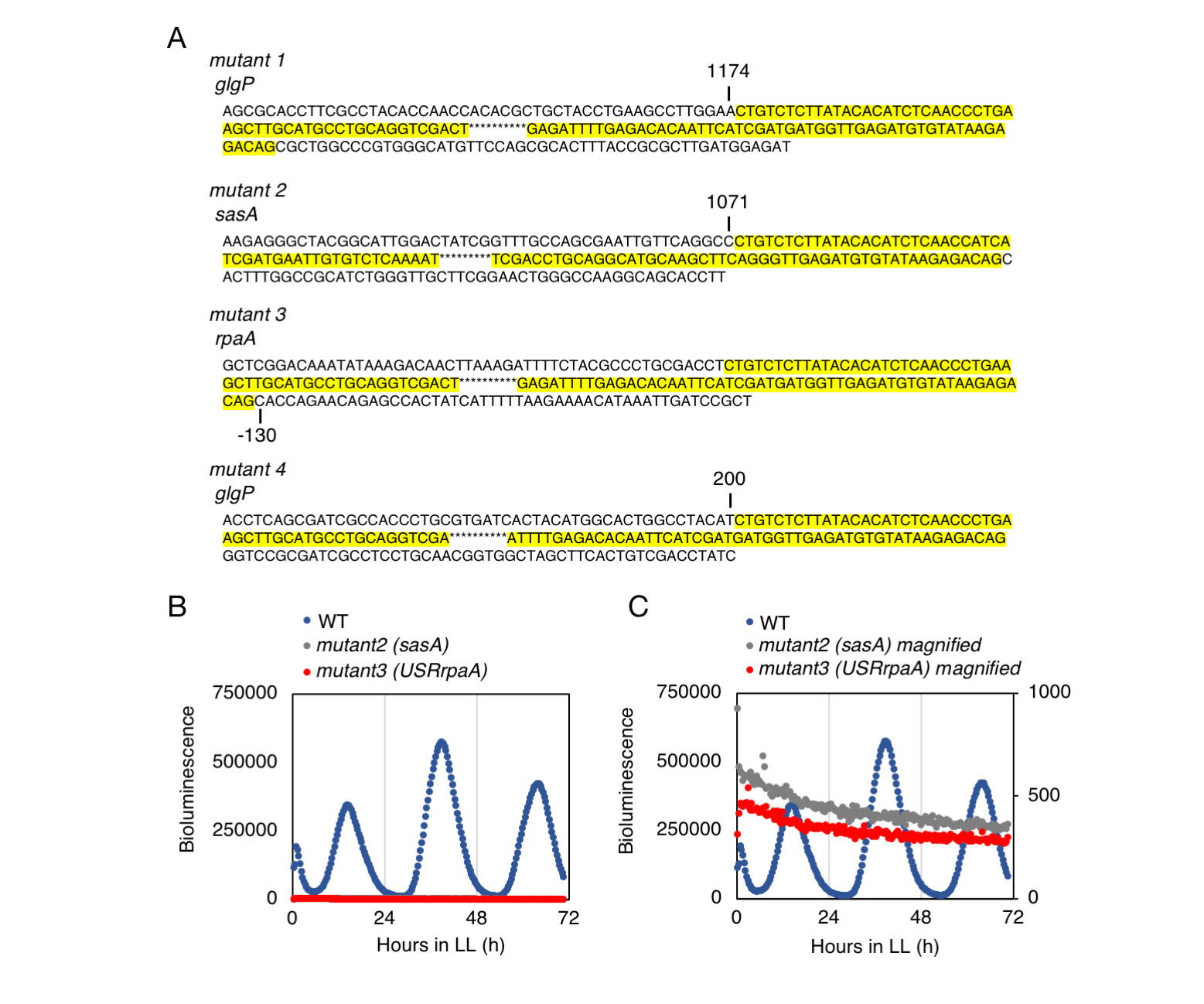

Supplement: S7 Fig — (A) Detailed sequences of Tn-5 insertion sites shown in Fig 4B. The intermediate sequence of the Tn-5 transposon was shown with asterisk (*) and only both ends of the transposon sequence are shown in yellow. The numbers indicate the positions of insertion sites when the initial nucleotide for the translational start codon of each ORF is assigned a positional value of 1. (B) Bioluminescence rhythms of WT, mutant 2 and mutant 3 cells that carried the PkaiBC::luxAB reporter cassette. The cells were grown on solid media under the LL condition after two LD cycles at 30 μmol/m2·s and bioluminescence were measured using photomultiplier tubes (n = 3). (C) For clarity, the bioluminescence profiles of mutants 2 and 3 are also shown in magnified scales (magnified scale shown on the right). (TIF) [file pgen.1009230.s007.tif]

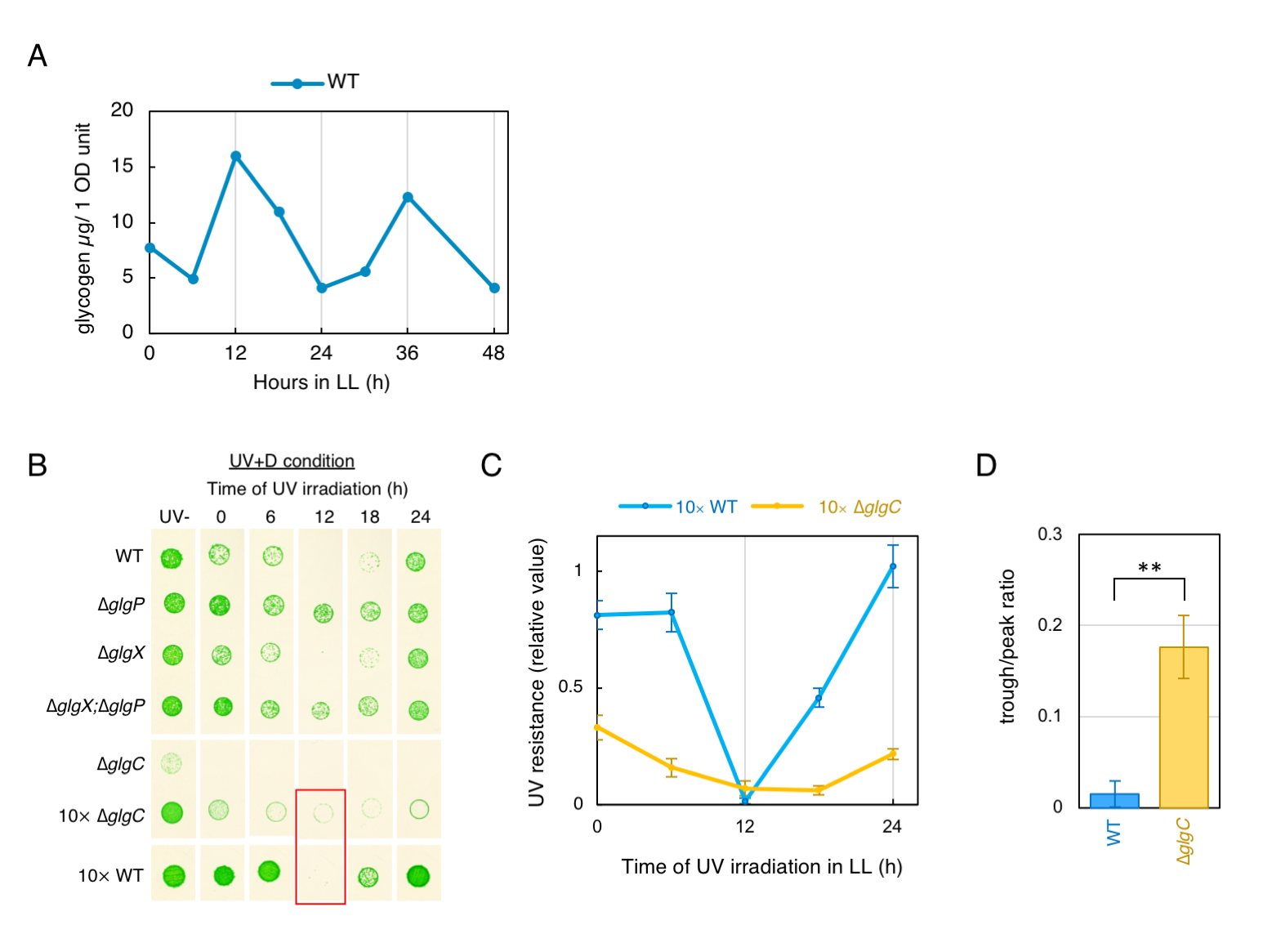

Supplement: S8 Fig — (A) Glycogen contents fluctuate in a circadian fashion under LL condition. Glycogen contents in WT were quantified. Hours in LL is shown on the horizontal axis, and glycogen content is shown on the vertical axis. The glycogen contents are normalized to the OD730 unit of harvested cells. (B) Effects of UV-C irradiation on glycogen-related mutants under the UV+D condition (ΔglgP, glgP-null mutant; ΔglgX, glgX-null mutant; ΔglgC, glgC-null mutant). Each image represents a spot assay to assess growth as shown in Fig 2B. It was difficult to evaluate UV-C resistance of the glgC strain due to dramatically lower viability under a 12-h:12-h LD cycle. Therefore, experiments were carried out with spots with ten-fold denser cell suspensions (ΔglgC and WT at the bottom). It should be noted that UV-irradiation at hour more severely suppressed colony formation in the WT cells than the glgC strain (red square), suggesting not only the magnitude of UV resistance but amplitude of the rhythm was greatly reduced in the mutant strain. (C) Densitometric analysis of the growth test in S8B Fig (ΔglgC and WT, spots with ten-fold denser cell suspensions). The timing of UV irradiation is shown on the horizontal axis, and relative UV resistance is shown on the vertical axis, as in Fig 1C (n = 3). Error bars represent standard deviation. (D) Trough to peak ratio of the UV resistance profiles in the wild type and glgC strains, using the maximal and minimal values of the UC resistance profiles shown in S8C Fig. The ratio significantly differs between the two species. **P< 0.01 (Student’s t-test). (TIF) [file pgen.1009230.s008.tif]
