# Supplementary material for: Involvement of glycogen metabolism in circadian control of UV resistance in cyanobacteria
Source: PLoS Genet. 2020 Nov 30;16(11):e1009230. doi: 10.1371/journal.pgen.1009230 (PMC7728383; doi:10.1371/journal.pgen.1009230)
Supplement: S1 Table — (DOCX) [file pgen.1009230.s009.docx]

**S1 Table. Strain used in this study.**

| Strain | Genotype | Host strain | Using plasmid for transformation |
| --- | --- | --- | --- |
| NUC42 | WT; P*_kaiBC_::luxAB* in NS I | - | - |
| ILC128 | ∆*kaiABC* | NUC42 | pDkaiABC (Ishiura *et al*., 1998) |
| ILC758 | ∆*kaiABC*; P*_kaiBC_::luxAB* in NS I | NUC42 | pDkaiABC (Ishiura *et al*., 1998) |
| ILC1124 | *kaiC^WT^*; P*_kaiBC_::luxAB* in NS I | ILC758 | pCkaiABC (Ishiura *et al*., 1998) |
| ILC1119 | *kaiC^A87V^*; P*_kaiBC_::luxAB* in NS I | ILC758 | pIL788 (for A87V mutant, this study) |
| ILC1122 | *kaiC^F470Y^*; P*_kaiBC_::luxAB* in NS I | ILC758 | pIL781 (for F470Y mutant, this study) |
| ILC665 | ∆*phr* | NUC42 | pIL762 (for ∆*phr* Gm^r^, this study) |
| ILC670 | ∆*phr;* P*trc::phr* in NS II | ILC665 | pIL770 (for introducing P*_trc_::phr* Km^r^, this study) |
| ILC779 | ∆*uvrA* | NUC42 | pIL847 (for ∆*uvrA* Sp^r^, this study) |
| ILC694 | ∆*glgP* | NUC42 | pIL791 (for ∆*glgP* Gm^r^, this study) |
| ILC773 | ∆*glgX* | NUC42 | pIL825 (for ∆*glgX* Sp^r^, this study) |
| ILC782 | ∆*glgP;* ∆*glgX* | ILC694 | pIL825 (for ∆*glgX* Sp^r^, this study) |
| ILC774 | ∆*glgC* | NUC42 | pIL826 (for ∆*glgC* Gm^r^, this study) |
| ILC1265 | ∆*sasA* | NUC42 | pAM2176 (Dong *et al*., 2010) |
| ILC1266 | *cikA^-^* | NUC42 | pAM2152 (Dong *et al*., 2010) |
| ILC626 | P*_trc_*::*glcP* in NS I | WT | pAL46 (McEwen *et al.,* 2013) |
|  |  |  |  |
