# Supplementary material for: Involvement of glycogen metabolism in circadian control of UV resistance in cyanobacteria
Source: PLoS Genet. 2020 Nov 30;16(11):e1009230. doi: 10.1371/journal.pgen.1009230 (PMC7728383; doi:10.1371/journal.pgen.1009230)

Comments to the authors:

The revised manuscript, “Involvement of glycogen metabolism in circadian control of UV resistance in cyanobacteria” by Kawasaki and Iwasaki, is much improved. The authors address our questions/concerns adequately except for our point about Figure 1, which we think can be improved for clarity. The figure in its current form implies that the plates were transferred (after the UV treatment) to regular growing conditions i.e. LL. Thus, to my eyes, it seems each plate did not undergo the full treatment (with all LD cycles). That is reinforced by the fact that the arrow (in the original panels A & D) that is shown for each plate is interrupted at the time that the plate receives the treatment, suggesting that that was an end-point and that they were withdrawn from the treatment then. Because the colonies are continued to be grown up after the UV treatment until they are quantified by the spot assay, I strongly suggest that the authors make the minor change to panels A & D of Figure 1 as shown here by adding the extra arrows:

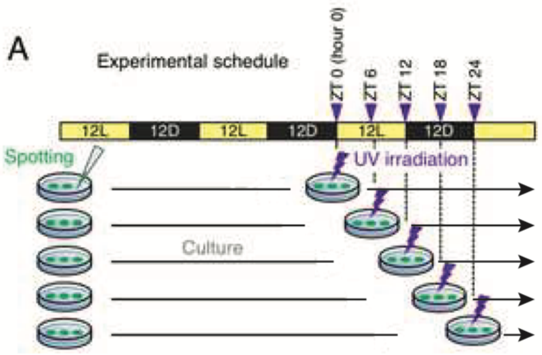

Supplement: Attachment — Submitted filename: Comments to authors.pdf [file pgen.1009230.s012.pdf]
