# Supplementary material for: Involvement of glycogen metabolism in circadian control of UV resistance in cyanobacteria
Source: PLoS Genet. 2020 Nov 30;16(11):e1009230. doi: 10.1371/journal.pgen.1009230 (PMC7728383; doi:10.1371/journal.pgen.1009230)
Supplement: Attachment — Submitted filename: Response_to_Reviewers20201008.docx [file pgen.1009230.s011.docx]

One-by-One Response to Reviewers

*Text in *italic* is a quote of the comments from the reviewers.

*Reviewer #1: The paper by Kawasaki and Iwasaki reports a study of a circadian-clock mediated response to UV light in the unicellular cyanobacterium Synechococcus elongatus. The authors found expression of an activity of resistance to UV light dependent on central clock components (Kai proteins). Importantly, the resistance is hidden by the very active DNA photolyase of cyanobacteria, which made the authors to carry out an exhaustive genetic analysis to be able to demonstrate the phenomenon. The expression of resistance is higher at dawn, preparing the organism to fight UV during daytime. This is an important finding. The authors then carried out transposon mutagenesis and identified genes participating in the circadian UV-resistance response. They found circadian clock-related genes, confirming the original finding, and mutations in gene glgP encoding a glycogen degradation enzyme. Complementary experiments allow the authors to propose that UV-resistance is counteracted by active metabolism through an unknown mechanism. The paper is well written and the experiments described are sound. I have only a few formal points:*

We thank the reviewer for careful reading of the manuscript, comments and suggestions.

*1. The last paragraph of the introduction is very reiterative with the abstract. You may reduce it significantly.*

According to this and other reviewer’s suggestion, we modified the introduction to reduce overlapping (lines 52-94)

*2. Lines 121-122: It is hard to evaluate how significant this difference is.*

According to this suggestion, we added the statistical result to the legend to Figure 1C as follows.

“Error bars represent standard deviation. The UV resistance in the WT and ∆*kaiABC* strains at ZT 18 significantly differ. **P* < 0.01 (Student's t-test).” (line 777-778)

*3. Line 149: 60 hours (“hours” missing).*

We fixed the error. (line 149)

*4. Line 159: please indicate the time units for UV-B irradiation in Fig. S3A.*

We added the time unit to S4 Fig (previously Fig S3A).

*5. Line 197: “The photorepair reaction is…”, not “are”.*

We fixed this error. (line 193)

*6. Line 199: please spell out CPD.*

We fixed this as follows:

“Major DNA damage caused by UV irradiation results in cyclobutane pyrimidine dimers (CPDs) formation [24, 25].” (lines 195-196)

*Reviewer #2: The manuscript, “Involvement of glycogen metabolism in circadian control of UV resistance in cyanobacteria” by Kawasaki and Iwasaki, is a worthy addition to the literature on circadian rhythms in cyanobacteria, with important implications for daily rhythms of resistance to UV light that have been hypothesized to underlie the early evolution of circadian rhythmicity. There are a number of issues that the authors must address in a revised manuscript:*

We thank the reviewer for very thoughtful and valuable suggestions, comments and questions.

*1. Abstract (line 49): remove the phrase “through unknown mechanism(s).” These three words make the Abstract sound weak and they are unnecessary because this issue is thoroughly addressed in the DISCUSSION.*

We understood the point. We erased the sentence and revised the corresponding text as follows:

“Combining these results with further experiments using ATP synthesis inhibitor and strains with modified metabolic pathways, we showed that UV-C resistance is weakened by directing more metabolic flux from the glycogen degradation to catabolic pathway such as oxidative pentose phosphate pathway and glycolysis.” (lines 45-47)

*2. Fig. 1B/E and C/F: given that the LD and LL protocols are the same for the first 12 hours after the beginning of the UV exposures, the UV resistances SHOULD be the same for hours 0 & 6 (and 12 ?, see next point) in panels B/C versus panels E/F. Why aren’t they?*

In Fig. 1B/E cells are exposed to the dark for 12 h (at the timing of ZT12) after UV irradiation, while in Fig. C/F cells are NOT exposed to the dark at any time after UV exposure. We suggest this difference in the experimental schedule caused the difference in the results. To clarify this, we added the text as follows:

“Even though the conditions before the time of UV exposure at 0, 6 and 12 hours were the same for both LD and LL conditions, the results of the growth test were different. This is because that growth was affected by the presence or absence of the subsequent darkness.” (lines 112-115)

*3. Fig. 1B/C: for the “12” and “24” hour timepoints, was the UV exposure initiated just before or just after the dark/light transitions?*

UV exposure was initiated just before the dark/light transitions. We have added more detailed information in Materials and Methods.

“UV exposure was initiated just before the dark/light transitions under diurnal condition (ZT = 0, 12, 24) and UV+D condition.” (lines 505-506).

*4. Fig. 1 legend: what does “three independent experiments” mean? Does it mean three experiments with an n of 1? Or three experiments with multiple replicates each? Or do the authors actually mean three triplicates within one experiment performed at the same time?*

Data we quantified in the paper were obtained the results of experiments at the same time using independently cultured cell suspension. In addition, we have confirmed that at least two or more other independent experiments performed at different timing yielded essentially same results. The reason why we showed data obtained from the experiments performed at the same time in the paper, however, is to eliminate subtle background difference in growth condition.

“The quantified densitometric value (*n* = 3) were obtained from experiments performed at the same time using three independently pre-cultured cells. We have confirmed that at least two or more completely independent experiments (independently precultured cells to be analyzed on different plates at different time) obtained essentially similar results.” (lines 516-520)

*5. Fig. 2A/B/C: is the UV- control a culture that is maintained in LL the entire time? From the perspective of optimal experimental design, there should be UV- controls that experienced the 6h dark exposures at each timepoint. If that experiment has not been done, the authors need to do it.*

We thank the reviewer’s suggestion. According to the reviewer's suggestion, we performed additional experiments to confirm dark exposure only is insufficient to cause time-dependent variation in growth. We irradiated the cells with dark only and UV+D at different timepoints (hours 0, 12, and 24 in LL), and compared the growth of the cells. Cells exposed to darkness without UV did not show any time-dependent attenuation of growth, while the growth was severely restricted when cells were exposed to the dark immediately after UV irradiation at subjective dusk (hour 12 in LL). In addition, it should be noted that a number of papers, including ours, on *Synechococcus* have reported on the phase response curved against dark pulses (Schmitz *et al.* Science 2000; Kiyohara *et al*., J. Bacteriol. 2005; Umetani *et al.*, J. Bacterial 2014). We have not observed growth defects by short dark pulses administrated at any time during the circadian cycle, while longer (~18-h) darkness is known to cause slight change in the speed of cell elongation when cells were exposed to the dark at either dawn or dusk (Lambert *et al*., Biophys J. 2016). Thus, we added newly prepared S2 Fig and changed the text as follows.

“The “UV-”control samples shown in Figs 2A-C were cultured under LL conditions after entraining the clock to two LD cycles. We confirmed that the time-dependent variation of growth was not caused by darkness alone, as shown in S2 Fig. When the growth of cells was compared under UV+D condition or 6-h darkness only condition without UV at different time (0, 12, and 24 h in LL), dark exposure for 6 h alone did not cause time-dependent difference in growth, whereas UV+D at hour 12 in LL significantly reduced.” (lines 127-132)

*6. Fig. 2D: the test with short period mutants is excellent, but the conclusion would be strengthened if the authors also show equivalent data for a long-period mutant.*

We understand the point. However, we believe the current data are already strong enough to lead the conclusion. In addition, if long period mutant strains are used, the time interval between giving UVs will be longer. This would mean that we would be treating cells at different growth phases, which may add noise that is not circadian clock-dependent but growth phase dependent to the results. Therefore, we do not think this experiment necessary, especially under this restricted lab conditions, under which we cannot do experiments from 22:00-9:00 in our university.

*7. Fig. S2: the data for liquid culture only covers 1 cycle in LL, while for the spotted cultures, the researchers tested 2 cycles (Fig. 2). It would be good to do 2 cycles in the liquid cultures also to confirm a self-sustained rhythm.*

We present the data on the liquid culture system to confirm that the experiments obtained by the spot test can be reproduced by other methods. Self-sustainability of the rhythm has been repeatedly validated with the spot test, and we believe that the conclusion has been sufficiently shown already without any question.

*8. Lines 164-165: which figure are the authors referring to?*

We have fixed this point as follows:

“Importantly, in the Δ*kaiABC* strain, the rhythm of UV resistance was abolished with constitutively lower levels (approximately trough levels of the WT strain, Fig. 2C).” (lines 163-164)

*9. Line 197: "are" should be "is"*

We have corrected this error (line 193).

*10. Page 8: define “CPD” (Cyclobutane Pyrimidine Dimer). I had to look it up because this abbreviation is not defined in the manuscript.*

We are sorry for having bothered the reviewer. We fixed the point. (line 195)

*11. Fig. 3B: show histograms for the UV- control data. The way the data are currently presented imply that there was no control.*

It is not the case. In the previous manuscript, all data shown in Fig. 3B were already normalized to the UV- control data, as noted in the legend to the figure. To make this point clearer, we have modified the vertical label in Fig.3B, so that normalization to the UV- control data was more emphasized. The values for the control are provided in the source data file. (Figure 3B)

*12. Fig. S4F/G: From the authors’ previous microarray studies, was UVR rhythmically expressed? I’m not clear on the authors’ logic here: if the deletion of UVR causes the loss of rhythmic resistance in the UV+D protocol (Fig. S4G), isn’t that result consistent with rhythmic regulation of UVR being responsible for the results shown in Fig. 2?*

We thank the reviewer’s questions. The *uvrA* mutant reduced the magnitude of UV resistance under UV+D condition, while it still remains a residual circadian rhythm. However, we are sorry for the reviewer that in the previous pdf file, the photo of *uvrA* mutant in S5G Fig (previously Fig. S4G) was unclear so that the rhythm was not evident. Thus, we have added quantified data (S5HI Figs). Since *uvrA* strain did not completely abolished the rhythm, we suggest that *uvrA* is not functioning as a major factor to drive the UV resistance rhythm. We have added the *uvrA* mRNA profile in our previous microarray analysis as shown in S5J Fig, according to the reviewer, and largely modified the text as follows:

“Nucleotide excision repair (NER) is a representative photorepair-independent DNA repair mechanism. Inactivation of the *uvrA* gene, which participates in the NER process, moderately reduced UV-C resistance to ~50% of that in the wild type strain under the UV+L condition (S5F and S5H Figs). More significantly, UV-C resistance was dramatically lowered in the *uvrA* strain under the UV+D condition (S5G and S5I Figs). Nevertheless, an attenuated time-dependent variation in UV-C resistance still remained (S5I Fig, see the magnified line), suggesting that *uvrA* is important for the magnitude of UV resistance but not essential to drive the rhythmicity. Our previous DNA microarray analysis indicated that the *uvrA* mRNA expressed in a *kai*-dependent circadian fashion as many other genes (S5J Fig, [14]). However, its amplitude is low with peak-to-trough ratio of ~1.4, which would be insufficient to drive a high amplitude protein abundance rhythm. Although we cannot rule out the possibility that clock-controlled NER somewhat supports and amplify UV resistance rhythms when CPD photolyase activity is partially inhibited by dark exposure, further studies are needed to validate this possibility.” (lines 245-256)

*13. Fig. 4: please give specific information as to the location and identity of Mutants 1-4 (e.g., provide insertion sites and the transposon; perhaps adding to the Supplement the sequence of the transposon with 50 nucleotides of the adjacent gene sequences on either side of the transposon sequence would suffice for each Mutant). Other researchers may want to repeat your results.*

According to this suggestion, we added more detailed information on the Tn-5 insertion sites in newly prepared S7A Fig with the legend as follows:

“(A) Detailed sequences of Tn-5 insertion sites shown in Fig. 4B. The intermediate sequence of the Tn-5 transposon was shown with asterisk (*) and only both ends of the transposon sequence are shown in yellow. The numbers indicate the positions of insertion sites when the initial nucleotide for the translational start codon of each ORF is assigned a positional value of 1.” (lines 951-954)

*14. Fig. 4: Puszynska and O’Shea reported (2017 eLife) that ΔrpaA has a lower glycogen content compared with WT, but why didn’t Mutant 3 in rpaA (Fig. 4A) show a lower resistance to UV?*

We thank the reviewer’s comment. This is very important question, which another reviewer also asked. We largely modified the text to answer this question as follows:

“Interestingly, glycogen content was lower in the *rpaA* strain, despite the fact that glycogen synthesis activity was kept high in the *rpaA* strain [37]. Although it is not fully understood why glycogen synthesis activity remains high and subsequent glycogen synthesis is restricted. However, it can be understood without contradiction if one considers that the intracellular state of the *rpaA* strain is arrested in the dawn phase state of the wild strain. When cells are arrested in the dawn phase, metabolic flows including carbon sources may not operate since resources for glycogen synthesis are depleted. This background indicates that it is not easy to estimate the intracellular physiological state, including the activity of glycogen degradation and synthesis, based solely on the amount of glycogen content at each time. The link between UV resistance and glycogen metabolism suggested by the screening would be either the result of the glycogen content itself, the effect of the activity of glycogen degradation by *glgP*, or the effect of the downstream pentose phosphate circuit and the TCA cycle.” (lines 321-331)

*15. Fig. 5: Has the glgC gene been overexpressed to assess whether the glycogen content is altered and its effect on UV resistance?*

We have not tested it. However, our data in single or double mutant strains deficient of *glgC, glgP* and/or *glgX* have already supported that genes related with glycogen metabolism modify UV resistance profile. Moreover, we have shown that the glycogen content was actually altered in the *glgP* strain, which dramatically altered UV resistance profile (Fig. 5B). Based on these and other data shown in the manuscript, we believe that the relationship between glycogen metabolism and UV resistance is sufficiently discussed even without *glgC* overexpressor. In addition, in general, overexpressed strains are often found to produce unexpected secondary effects. To avoid the uncertainty of such effects, in a previous manuscript we showed correlation between UV resistance and downstream of the glycogen catabolic pathway, and UV resistance profile using a more characterized metabolically altered (glucose-uptake) strain (Fig. 5E). The pathway into which a given carbon source (glucose) flows in this strain has already been elucidated by metabolomic analysis (Kanno et al., 2017, 10.1038/ncomms14724). To address these points, we modified the text as follows. We believe this rewording much helps the readers for better understanding.

“In order to validate whether changes in further downstream catabolic pathways affect UV-C resistance, we used a strain that was genetically modified to uptake exogenous glucose directly into the cells, as reported previously [40]. It should be noted that we also considered another possibility to use strains overexpressing the *glgP* and *glgC* genes to activate glycogen catabolism. However, overexpression strains may cause unexpected side effects. More importantly, it is obscure whether overexpression of metabolic enzymes really activates the magnitude of targeted metabolic pathways, considering the amount of the carbon source itself may not change. On the other hand, it has been shown in the forced glucose uptake strain that glucose is incorporated into OPPP and the Emden-Meyerhof pathway (EMP) to be used as *bona fide* carbon source (Fig 5E, [41]). In this strain, the *glcP* gene, which encodes a glucose transporter derived from another cyanobacterium, was expressed by the *trc* promoter in the presence of IPTG.” (lines 396-405)

*16. Line 383: “contrast” is misspelled.*

We fixed the point.

*Reviewer #3: This is a very interesting manuscript that links UV sensitivity to oxidative phosphorylation in cyanobacteria.*

*The authors characterize the circadian control of UV sensitivity in the cyanobacterium Synechococcus elongatus PCC. They demonstrate that the UV sensitivity changes throughout the day with a maximum at dusk under light/dark cycles. They make the observation that when cells are maintained under constant light there are not cyclic changes in UV sensitivity, but that a dark period after the UV treatment reveals cyclic sensitivity. Using mutants of DNA repair enzymes, they demonstrate that the cyclic sensitivity does not seem to be strongly regulated by either the photolyase or enzymes involved in nucleotide excision repair. The authors argue that the ‘necessary’ dark period after the UV treatment inhibits the photolyase activity revealing the cyclic sensitivity under constant light conditions. The authors also screened a mutant population for lines with high resistance to UV at dusk. They discovered mutations in circadian clock components that lock gene expression in a ‘dawn’ state, which has an associated high UV resistance. In addition, they identified two lines with mutations in a glycogen-phosphorylase gene. Further experiments demonstrate that consumption of glucose via oxidative phosphorylation in the dark is associated with high UV sensitivity.*

*The manuscript is clear and well written the results presented will be relevant to researchers working on UV sensitivity in all photosynthetic organisms, as well as researchers working on the role of mitochondria on oxidative stress.*

We thank the reviewer for thoughtful and valuable suggestions and comments.

*1. I think their results link UV to mitochondrial oxidative phosphorylation, and glycogen metabolism is just an example of a more general principle. I elaborate on this point below.*

We answered to more detailed comments and suggestions one by one below.

*2. Similar results of the ‘dark’ requirement for circadian controlled sensitivity were described in land plants by Takeuchi et al.(2014) (doi:10.1093/jxb/eru339). Although the authors cite the work of Feher et al, 2011, these authors only report UV mediated control of gene expression and not sensitivity, which did not appear to be circadian controlled in constant light, as observed in cyanobacteria. Takeuchi et al.(2014) demonstrated circadian controlled UV sensitivity that is darkness dependent in the model plant Arabidopsis thaliana, carrying experiments analogous to the ones reported here. This does not minimize the importance of the current study, on the contrary, it shows that the phenotype and mechanism reported might be widely relevant in photosynthetic organisms.*

We thank the reviewer for this suggestion. Although Takeuchi *et al.* did not discuss our proposal for dark-exposure for unveiling masking effect by photolyase activity, we agree to the point, and added the text as follows:

“In *Synechococcus*, the presence of darkness after UV irradiation significantly changed its time dependent UV sensitivity. It is similar to the daily changes in UV-B sensitivity observed in *Arabidopsis thaliana* [42]. In *Arabidopsis*, UV-B sensitivity is high in subjective dusk only when exposed to darkness after UV-B irradiation. It should also be noted that there is difference in experimental schedule between the two studies: in *Arabidopsis*, pre-UV irradiation with lower light intensity was applied a few hours before the UV-B irradiation, while such pre-irradiation was not necessary for our experiments. Nevertheless, the similarity suggests a common property in the circadian control of UV resistance across species.” (lines 417-424)

*3. There are a couple of instances in which I don’t agree with the written description of the results. These discrepancies don’t have a big impact on the main conclusions of the paper but should be corrected. More details are below.*

*(1)The link with oxidative phosphorylation: I believe the authors should change the title to ‘Involvement of mitochrondrial oxidative phosphorylation in circadian control of UV resistance in cyanobacteria’. The authors show that the availability of carbon for respiration is linked to increased sensitivity to UV at dusk. This effect is not necessarily glycogen dependent, since feeding the cyanobacteria glucose in the dark has the same effect.*

We do not agree to changing the current title to the suggested one. First of all, one simple reason is that there are no mitochondria in cyanobacteria. In cyanobacteria, the electron transfer system is partly shared by the respiratory chain and photosynthetic machinery, different from eukaryotic plants. This makes it difficult to independently discuss oxidative phosphorylation activity from reactions in photosynthesis in cyanobacteria. However, as the reviewer suggested, change in glycogen content itself is not essential for altering UV resistance, because it was able to be mimicked by ectopic shift in their downstream metabolic activity, such as glucose feeding. However, in the wild type strain, it is likely that rhythmic activation of glycogen metabolism triggers metabolic effects on its downstream such as glycolysis and OPPP pathway. Thus, we still believe rhythmic change in glycogen metabolism is the key process for UV resistance rhythm.

*In addition, the authors demonstrate that the inhibition of oxidative phosphorylation suppresses UV sensitivity at dusk. Under constant light, oxidative phosphorylation is not critical for the cells since they are photosynthesizing. It seems that the correlation of oxidative phosphorylation with DNA synthesis leads to high sensitivity to UV. Since the bacteria cells expressing the glucose transporter can be grown under constant darkness in the presence of glucose, the authors could further test whether the circadian control occurs in constant darkness.*

We agree that suggested experiment would be interesting if it works well. However, there are several difficulties in the suggested experiment, and we disagree with the experiment. First of all, the wild type strain cannot grow in complete darkness. Therefore, the phenotype obtained under continuous dark conditions will only be observed in strains with modified metabolic pathways. As shown in Fig. 5E, that strain already showed a completely different UV resistance profile than WT. We believe that this is due to an altered and metabolic pathway partially arrested at an activated state. In other words, even if we were able to measure the UV resistance profile in this strain in continuous darkness, we would not be able to compare its profile to that of the wild strain, making it difficult to verify the mechanism. The second point is that if we were to apply UV irradiation under DD conditions, the post-UV period in the dark should last longer, and the survivability of cells must be attenuated. This is predicted by the fact that the longer the dark period after UV irradiation, the worse the growth of the cells becomes. Indeed, almost no cell survival has been observed when the dark period after UV irradiation last for about 12 h (see S4 Fig (previously S3 Fig) and Fig .1BC). In addition, under DD conditions, the photolyase activity will be continuously inhibited, which would be similar to that of the *phr*-deficient cells in LL, which causes serious effects on UV resistance in both UV+L and UV+D conditions, as shown in the manuscript.

*Are rhythms of oxygen consumption in the dark correlated with changes in UV sensitivity?*

To our knowledge, oxygen consumption has not been tested in this species in DD. Although the level of dissolved oxygen in *Synechococcus* showed a clear diurnal rhythm under LD cycles, with a sharp increase in respiratory activity immediately after the onset of the dark exposure at ZT 12, the rhythm was immediately damped out in LL (Yen et al., 2004, https://doi.org/10.1016/j.plantsci.2003.12.005). Therefore, it seems difficult to discuss the magnitude of oxygen consumption and the UV resistance rhythm for this moment. Instead, we have performed additional experiments using DBMIB, which specifically inhibits the electron transfer in the respiratory chain as shown below (for reviewers only). If we look very carefully the data, the effect of UV-C irradiation may be slightly attenuated when the electron transfer in the respiratory chain is inhibited by DBMIB. However, we have not obtained data which differs statistically significant to the control. Thus, to avoid confusion this result has not been included to the revised manuscript.

*In addition, I don’t fully understand the relationship between SasA, RpaA, glgP and glycogen content. The loss of SasA leads to low glgP expression (Fig. 4). The loss of glgP leads to a decrease of glycogen degradation in the dark. But why is the glycogen content in the SasA mutant lower than the wild type at dusk as reported previously? Could the authors confirm that result?*

We thank the reviewer’s comment. This is very important question, which another reviewer also asked.

We largely modified the text to answer this question as follows:

“Interestingly, glycogen content was lower in the *rpaA* strain, despite the fact that glycogen synthesis activity was kept high in the *rpaA* strain [37]. Although it is not fully understood why glycogen synthesis activity remains high and subsequent glycogen synthesis is restricted. However, it can be understood without contradiction if one considers that the intracellular state of the *rpaA* strain is arrested in the dawn phase state of the wild strain. When cells are arrested in the dawn phase, metabolic flows including carbon sources may not operate since resources for glycogen synthesis are depleted. This background indicates that it is not easy to estimate the intracellular physiological state, including the activity of glycogen degradation and synthesis, based solely on the amount of glycogen content at each time. The link between UV resistance and glycogen metabolism suggested by the screening would be either the result of the glycogen content itself, the effect of the activity of glycogen degradation by *glgP*, or the effect of the downstream pentose phosphate circuit and the TCA cycle.” (lines 321-331)

*The authors also need to be more careful and precise when discussing the role of metabolism. For example, on line 364 they appear to argue that ‘de novo energy production via glycogen degradation in the dark’ is involved in the changes in sensitivity. The cells produce a lot of ATP during the light period without any apparent negative effect, so ATP production per se is not an issue, however mitochondrial oxidative phosphorylation appears to be the link to increased sensitivity. In line 439 the authors argue that high UV resistance would antagonize energy production process. Not necessarily, since that is not a problem in the light. I would rather conclude that under carbon limitation in the dark a decrease in UV-resistance might provide a fitness advantage. However, it is also possible, that mitochondria might be more susceptible to UV stress when oxidative phosphorylation is high due to the production of reactive oxygen species.*

We thank the reviewer’s thoughtful comment and suggestion. As pointed out by the reviewer, although ATP production is plentiful in the light (e.g. the WT strain under UV+L condition), UV resistance does not seem to be low. We believe that the higher UV resistance in light is due to the higher DNA-damage-repairing activity by the photolyase, which masks the effects of UV irradiation. As shown in S5C Fig (previously S4C), the *phr* strain shows extremely higher sensitivity to UV under UV+D condition. It can be interpreted as indicating increased UV sensitivity when the photolyase activity is suppressed (just as in darkness) but with highly activated state of ATP production. As for possible link between ATP production and UV resistance, we suggest a harmful effect of DNA replication process using UV-damaged DNA as a template. The absence of mitochondria in cyanobacteria is mentioned above. To address these points, we added the text as follows:

“Although ATP production is highly active in the light, UV resistance does not appear to be low, as shown in the WT strain under UV+L conditions. We believe that the higher UV resistance in the light is due to the higher DNA damage repair activity of photolyase, which masks the effects of UV irradiation. The very high UV sensitivity in the photolyase deficient strain under UV+L condition (S5C Fig) can be interpreted that this strain is highly sensitive to UV in the light when ATP production is highly active without being masked by the photolyase activity.” (lines 388-393)

In addition, we also modified the related text in Discussion as follows:

“The observations using CCCP treatment and forced incorporation of glucose (Fig 5) are consistent with our hypothesis that increased catabolic flux (from glycogen degradation to the downstream OPPP and glycolytic pathways) at subjective dusk causes lowered UV resistance, although further analysis is necessary to reveal the molecular mechanisms underlying the metabolism-gated, circadian UV-resistance system. It should be noted that in *Synechococcus* the rate of DNA synthesis is not under circadian control in LL but oscillates under LD condition [43]. This is because DNA replication of cyanobacteria stops after cells are exposed to the dark. However, the DNA replication activity immediately after dark exposure changes due to glycogen degradation activities in the dark. When glycogen degradation activity is relatively higher after cells are transferred to the dark, DNA replication persists with a longer duration due to elevated abundance of ATP-bound DnaA[44, 45]. Since glycogen degradation activity of *Synechococcus* seems to increase at dusk, DNA replication continues for a while even after the onset of dark acclimation. If this is the case, UV irradiation and transfer to darkness (UV+D condition) at dusk, but not at dawn, would result in unstable replication of damaged DNA without photolyase-mediated repair. In *E. coli*, it is known that double strand breaks occur when the replication fork hits unrepaired DNA, resulting in lethality [46].” (lines 437-450)

*In the abstract and the discussion the authors also argue that glycogen degradation or ‘increased metabolic flux’ is required for the increased UV sensitivity. That is actually not the case as their nice experiments using the glucose feeding demonstrate. ‘Metabolic flux’ is an ambiguous term in this context.*

We thank the reviewer’s comment. We agree that the term 'metabolic flux' was too ambiguous. Thus, we have changed it to be more specific in the revised manuscript. The term 'metabolic flux' in the revised manuscript assumes a catabolic pathway starting from glycogen degradation to the downstream oxidative pentose phosphate cycle and the glycolytic system as represented in Fig. 5A. To clarify this point, we have added this point everywhere 'metabolic flux' was mentioned. (lines 46, 89 and 438).

*(3) The written description of the results does not seem to match the figures in the following examples:*

*a. Line 255: ‘inactivation of uvrA gene, […], had not effect on UV-C sensitivity under the UV+L condition’. Fig. S4F demonstrates that this mutant was more sensitive than the wild type in UV+L and UV+D, although the effect was stronger in UV+D. In this and other cases, would be clearer to show the quantification of the cell density analysis with the necessary replicates and statistics instead of showing only the images.*

We agree to this suggestion, and added quantified data with replicates and statistics as S5H and S5I Figs.

*b. The authors did not measure glycogen levels in the glgX mutant strain, they cannot conclude its effect on glycogen based on the effect of this mutation of UV sensitivity. The experiments shown in Fig. S7B prove the effect of glgP in UV-C sensitivity not in glycogen metabolism, since no glycogen was measured.*

We did NOT observe any significant changes in the UV sensitivity of *glgX* mutants (S8B Fig). On the other hand, we showed the effect of *glgP* nullification on UV-C sensitivity. Despite the reviewer’s comment, we had already mentioned about the glycogen accumulation profile of *glgP* mutants in Fig. 5B. In fact, the *glgP* strains showed maintained glycogen levels during in darkness starting at dusk, while glycogen level was more reduced in the WT strain (Fig. 5B).

*c. Line 353: the authors state that in the glgC mutant strain UV-C resistance was decreased constitutively (Fig. S7B), however the 10x experiments demonstrate that that is not the case, at least based on the figure shown. The cell/colony density should be quantified and the authors should carry out proper statistical analyses.*

We agree to this suggestion, and added S8C Fig for quantified data with replicates. We also added S8D Fig to compare the peak/trough ratio of the rhythm to show reduced amplitude in the *glgC* strain.

*Other comments:*

*Figure S4C is mentioned before S4A and B in the text. The authors might want to change the order of the labels for clarification.*

We thank the reviewer for this comment, and fixed it. (Previous S4 was renamed S5, lines 203-205)

*Is Figure 3A the same as Figure S4D?*

That is essentially correct, thought they are from independent experiments. S5D Fig (previously S4D) was added in order to be compared with S5E Fig (in the presence of IPTG). To avoid confusion, we added the text to the legend as follows:

“S5D Fig and Fig. 3A are based on the same experimental design, while the images were obtained from independent experiment as the IPTG- negative control against the IPTG+ data shown in S5E Fig.” (S5 Fig Legend, lines 926-928)

*The authors define the term zeitgeber (ZT), it would help using it throughout the text and figures to better unambiguously define the time. For example, line 273, the authors write ‘at hour 12’ which is ambiguous.*

We thank the reviewer on this comment. Since ZT is used for regular LD cycles, we did not use this term for our UV+D or UV+L conditions. To avoid confusion, instead, we added our definition of “hour 0” in each experiment on Figs 1A, 1D, 2A and 3A (in any cases, time of light onset after two LD cycles to reset the clock). In addition, as for previously line 273 was modified as follows:

“when UV was irradiated at hour 12 in the light under the UV+D condition (Fig. 2A, see Materials and Methods, for experimental schedule).” (lines 269-270)

*The authors should mention that DNA synthesis occurs at dusk in Synechococcus (Mori et all, 1996) in both LD and LL.*

We understood to add info on DNA synthesis is important. It should be noted that in LL Mori *et al.* demonstrated DNA content per cell “peaks early subjective night phase and troughs in the late subjective-day phase”, while they also mentioned about the rate of new DNA synthesis that “the average slope of zero, suggesting that DNA synthesis initiates and proceeds at a constant rate, even though the cells are dividing rhythmically” (in LL).

In addition, Ohbayashi *et al.* (PCP 2017) reported DNA synthesis is reduced in the dark in relation with glycogen metabolism, as was previously described at the initial submission. Based on these reports and newly added related reference on the study in *E. coli*, we added and modified text as follows:

“It should be noted that in *Synechococcus* the rate of DNA synthesis is not under circadian control in LL but oscillates under LD condition [43]. This is because DNA replication of cyanobacteria stops after cells are exposed to the dark. However, the DNA replication activity immediately after dark exposure changes due to glycogen degradation activities in the dark. When glycogen degradation activity is relatively higher after cells are transferred to the dark, DNA replication persists with a longer duration due to elevated abundance of ATP-bound DnaA [44, 45]. Since glycogen degradation activity of *Synechococcus* seems to increase at dusk, DNA replication continues for a while even after the onset of dark acclimation. If this is the case, UV irradiation and transfer to darkness (UV+D condition) at dusk, but not at dawn, would result in unstable replication of damaged DNA without photolyase-mediated repair. In *E. coli*, it is known that double strand breaks occur when the replication fork hits unrepaired DNA, resulting in lethality [46].” (lines 440-450)

*Line 331: the authors state that the half-life of glycogen is ~ 6h. However, the authors did not measure glycogen at dawn, only during the first 6 h after dusk, so they cannot estimate its half-life. It is expected based on other experiments to be 6h, or better said, half of the dark period since cells are likely to optimize glycogen degradation, so that they don’t run out of carbon before dawn in a similar manner as starch in land plants.*

Contrary to the comment, we had measured the dynamics of glycogen degradation in the dark from dawn or dusk for 6 h as shown in Fig. 5B: The glycogen level fallen down dramatically to at least half of their initial value when darkness started from dusk (corresponding to half-life), while lesser degradation was observed in the dark starting from subjective dawn (Fig. 5B). The administrated darkness for 6 h mimics the post-UV dark exposure under the UV+D condition: it is important within the 6-h dark starting at either dawn or dusk differently affect on the glycogen consumption profile, consistent with different UV-resistance properties. However, we agree the use of the term ‘half-life’ may be confusing. So, we modified the text as follows:

“Glycogen is believed to be important as a carbohydrate reservoir, mainly for survival under ATP-limited dark condition in the absence of photosynthesis activity[36]. Therefore, we tested glycogen consumption profile during 6-h of darkness applied at subjective dawn and dusk. Dark exposure starting at hour 0 maintained glycogen content at constitutively low level (Fig 5B), comparable to the trough level of the circadian glycogen rhythm in LL (S8A Fig). On the other hand, dark exposure at hour 12 triggered more rapid glycogen degradation leading to ~45% within 6 h (Fig 5B). More importantly, ∆*glgP* strain did not show significant glycogen degradation during 6-h dark exposure, regardless of the time when the cells were exposed to darkness (Fig 5B). These results suggest that the higher UV-C resistance is accompanied by lower glycogen degradation activity, such as in the wild type strain at subjective dawn and in the *glgP* strain throughout the circadian cycle.” (lines 340-349)

*It is expected based on other experiments to be 6h, or better said, half of the dark period since cells are likely to optimize glycogen degradation, so that they don’t run out of carbon before dawn in a similar manner as starch in land plants.*

We thank the reviewer for this comment. However, it remains unclear to us if light-dependent optimization of degradation of glycogen is also the case in cyanobacteria: according to Diamond *et al.,* 2015 (DOI: 10.1073/pnas.1504576112), the linear degradation profile observed for starch in the land plant was not observed for glycogen in *Synechococcus*, suggesting somewhat different degradation dynamics between plants and cyanobacteria. So, we did not include that discussion.

*Line 361: I believe the authors mean ‘subjective dusk’ instead of ‘subjective dawn’.*

We corrected the error. (line 373)

*Line 483, please provide the intensity of the UV-B light use.*

We added the info onto Materials and Methods and the legend to S4A Fig as follows:

“UV-B irradiation was performed using UV transilluminator (PI-20.AB; BIO CRAFT, Tokyo, Japan) equipped with a discharge tube with a main wavelength of 312 nm. UV-B intensity was measured by a digital UV radiometer (Radiometer Sensor, UVX-31; Analytic Jena AG, Jena, Germany).” (lines 508-510)

“UV irradiation was performed using a discharge tube with a main wavelength of 312 nm (UV-B intensity was 5.22~5.88 mW/cm^2^).” (Legend to S4A Fig)

*More details about the mutant screen would be useful. How many cells were irradiated? How many initially ‘resistant’ colonies were found? After how many days did colonies form after irradiation?*

According to the request, we added more detailed information in Materials and Methods as follows:

“WT *Synechococcus* was transformed with a genomic DNA library fused to a Tn5-derived transposon with the kanamycin resistance gene [57] in order to introduce random mutations. This genomic DNA library was provided by Dr. Mitsunori Katayama of Nihon University (Tokyo, Japan). Transformants were streaked onto solid BG-11 plates containing kanamycin. After synchronizing the cells to two 12-h:12-h LD cycles, transformant cells were exposed to the light for 12 h, irradiated with UV-C as mentioned above, exposed to the dark for 6 h, and then returned to the light. Several days after UV irradiation, among 5,000 transformant clones screened, 18 colonies formed on the plates were initially picked up as possible mutants with greater UV resistance. Four out of the 18 clones were confirmed to show elevated survival rates after the UV+D treatment at ZT 12 by spot tests. All the four clones are reported in the present paper. Genomic DNA was extracted from each of the four mutant strains, and the transposon insertion sites were identified by inverse PCR and sequence analysis.” (lines 555-565)
