# Supplementary material for: Involvement of glycogen metabolism in circadian control of UV resistance in cyanobacteria
Source: PLoS Genet. 2020 Nov 30;16(11):e1009230. doi: 10.1371/journal.pgen.1009230 (PMC7728383; doi:10.1371/journal.pgen.1009230)
Supplement: Attachment — Submitted filename: Response_to_Reviewers20201027.docx [file pgen.1009230.s013.docx]

One-by-One Response to Reviewers

*Text in *italic* is a quote of the comments from the reviewers.

*Reviewer #2: Please see attachment, "Comments to authors.pdf"*

*The revised manuscript, “Involvement of glycogen metabolism in circadian control of UV resistance in cyanobacteria” by Kawasaki and Iwasaki, is much improved. The authors address our questions/concerns adequately except for our point about Figure 1, which we think can be improved for clarity. The figure in its current form implies that the plates were transferred (after the UV treatment) to regular growing conditions i.e. LL. Thus, to my eyes, it seems each plate did not undergo the full treatment (with all LD cycles). That is reinforced by the fact that the arrow (in the original panels A & D) that is shown for each plate is interrupted at the time that the plate receives the treatment, suggesting that that was an end-point and that they were withdrawn from the treatment then. Because the colonies are continued to be grown up after the UV treatment until they are quantified by the spot assay, I strongly suggest that the authors make the minor change to panels A & D of Figure 1 as shown here by adding the extra arrows:*

We fully agree to the reviewer’s suggestion. Not only additional extra arrows, we also add background color indicating light/dark regimen for further clarity. (Revised Figure 1)

*Reviewer #3: I first apologize for appear to indicate that cyanobacteria have mitochondria my previous comments to the authors, that is embarrassing. However, I think the authors understood my points which were related to oxidative phosphorylation and electron transport. The authors have now addressed all my initial questions and concerns.*

*I have just one short comment on the new paragraph that addresses the role of rpa in glycogen metabolism. The authors write:“ … glycogen content was lower in the rpaA strain despite the fact that glycogen synthesis activity was kept high in the rpaA strain”. I am not sure what the authors mean by ‘glycogen synthesis activity’. Puszynska and O’Shea measured the maximum catalytic activity of enzymes involved in the glycogen synthesis, not the actual flux towards starch, therefore I would change the wording to:“ … glycogen content was lower in the rpaA strain despite the fact that the maximum catalytic activity of enzymes involved in glycogen biosynthesis are not reduced in the rpaA strain”.*

We agree to this suggestion, and modified the text as instructed (see below).

*The authors also write:“When cells are arrested in the dawn phase, metabolic flows including carbon sources may not operate since resources for glycogen synthesis are depleted”. I am unclear what the authors mean by ‘metabolic flows including carbon sources’ and by ‘resources for glycogen synthesis are depleted’. The author need to clarify this point. Puszynska and O’Shea show that the energy charge is low at night in the rpaA strain indicating that this strain cannot regulate its energy balance efficiently, reacting strongly to light off (and on) conditions. Changes in ATP metabolism in the rpaA strain fit with the authors’ results using CCCP. A strong decrease energy charge at the beginning of the night (Puszynska and O’Shea) correlates with higher resistance in the rpaA strain. A strong drop in energy charge when the light goes off would also be expected in other mutants lacking glycogen that maintain high UV resistance. Why energy balance would affect UV sensitivity is the remaining open question.*

We understood the point. Although restoration of ATP production alone seems not sufficient for recovering glycogen metabolism, we strongly agree to the reviewer’s concern about ambiguous wording of metabolic flows and possible involvement of energy charge on this issue. Thus, including the former response, we modified the text as follows. We truly thank the reviewer’s thoughtful comment.

“Interestingly, glycogen content was lower in the *rpaA* strain, despite the fact that the maximum catalytic activity of enzymes involved in glycogen biosynthesis are not reduced the *rpaA* strain [37]. We suggest that the intracellular state of the *rpaA* strain is arrested at the dawn phase state of the wild strain, where glycogen synthetic enzyme may not catalyze its reactions since some steps for glycogen synthesis such as gluconeogenesis pathway are depleted. In the *rpaA* strain, energy charge (relative levels of ATP, ADP and AMP in the intracellular adenine nucleotide pool) strikingly drops when cells are exposed to the dark, compared with the wild type strain [37]. While restoration of energy charge by forced glucose uptake alone seems not sufficient to recover higher glycogen content [37], ATP synthesis may also be a rate-limiting factor for glycogen synthesis in the *rpaA* strain. In any cases, this background indicates that it is not easy to estimate the intracellular physiological state, including the activity of glycogen degradation and synthesis, based solely on the amount of glycogen content at each time.” (lines 336-346)

“These results are consistent with the hypothesis that *de novo* ATP synthesis in the dark after UV-C irradiation severely limited growth, and the circadian fluctuation in ATP availability via glycogen degradation would drive the rhythm in UV resistance. This is also consistent with the reduced energy charge in the *rpaA* strain when the cells are exposed to the dark [37], remaining higher UV resistance as discussed above.” (lines 404-407)
